# Cardiac Evaluation before and after Oral Propranolol Treatment for Infantile Hemangiomas

**DOI:** 10.3390/jcm13113332

**Published:** 2024-06-05

**Authors:** Ji Hee Kwak, Aram Yang, Hye Lim Jung, Hyun Ju Kim, Deok Soo Kim, Jung Yeon Shim, Jae Won Shim

**Affiliations:** Department of Pediatrics, Kangbuk Samsung Hospital, Sungkyunkwan University School of Medicine, 29 Saemunan-ro, Jongno-gu, Seoul 03181, Republic of Korea; jiheehihi.kwak@samsung.com (J.H.K.); aram.yang@samsung.com (A.Y.); dscw.kim@samsung.com (D.S.K.); jaewon.shim@samsung.com (J.W.S.)

**Keywords:** infantile hemangiomas, propranolol, cardiac evaluations

## Abstract

**Background:** Most recent clinical practice guidelines addressing the management of infantile hemangiomas (IHs) recommend oral propranolol, a non-selective beta-adrenergic antagonist, as first-line treatment. However, few reports have provided continuous follow-up data regarding cardiac evaluations. **Methods:** Sixty-four patients diagnosed with IHs and treated with oral propranolol before 2 years of age at the Department of Pediatrics, Kangbuk Samsung Hospital (Seoul, Republic of Korea), with regular examinations between 2017 and 2021, were included. Cardiac evaluations, including electrocardiography, Holter monitoring, chest X-ray, and echocardiography, were performed. **Results:** Sixty-four patients with IHs successfully underwent continuous follow-up cardiac evaluations. The median age at diagnosis was 2 weeks (1 day to 34.3 weeks). The median age at treatment initiation was 13.6 weeks (2.4–87.9 weeks), the mean longitudinal diameter of hemangioma at diagnosis was 2.8 ± 2.1 cm (0.3–12.0 cm), and the mean percentage of size decrease after 1 year of oral propranolol treatment was 71.8%. None of the 64 patients experienced severe adverse side effects during propranolol treatment. There was no statistically significant differences in echocardiographic function and electrocardiographic data after treatment. **Conclusions:** Propranolol treatment ≥6 months was effective and safe without significant cardiac toxicity in the treatment of patients with infantile hemangiomas.

## 1. Introduction

Infantile hemangiomas (IHs) are the most common benign vascular tumors among children, occurring in 5–10% of infants [1]. Pathologically, IHs are vascular neoplasms caused by a proliferation of abnormal endothelial cells and other vascular cells. Clinically, IHs are characterized by a rapid proliferative phase followed by a slow involutive phase. IHs are usually not present at birth but are diagnosed at 1–4 weeks of age, and rapidly proliferate between 1 and 3 months of age, with proliferation mostly complete by 5 months of age during early infancy [2]. IHs then spontaneously involute, mostly into adipose and fibrous tissue, until 4 years of age. Most IHs develop in the skin, including the epidermis, dermis, and subcutaneous fat of the head and neck (60%), trunk (25%), and extremities (15%), but rarely in the internal organs such as the liver, gastrointestinal tract, respiratory tract, heart, or brain [3]. The development of cardiac hemangiomas is rare, comprising 2.8% of primary cardiac tumors, with common sites including the right atrium and left ventricle. Cardiac hemangiomas are pathologically benign and proliferative endothelial cells that line the blood vessels with increasing vascularization and are histopathologically identical to those of IHs [4].

Due to spontaneous involution, most IHs can be actively observed for progression without treatment; however, 10–20% of IHs require early treatment due to location, size, and complications, including ulceration, bleeding, infection, and functional impairment. Clinical practice guidelines for the management of IHs published by the American Academy of Pediatrics recommend that primary care clinicians should frequently monitor infants with IHs, educate parents about the clinical course of IHs, and refer infants with high-risk IHs to IH specialists as early as 1 month of age. High-risk IHs indicated for early treatment include those with life-threatening complications, functional impairment, ulceration, associated structural anomalies, and disfigurement. Since 2015, management guidelines for IHs have been published in the Unites States, Europe, Australia, Japan, and Korea [2,5,6,7,8], and recommend oral propranolol (i.e., an oral, non-selective, beta-adrenergic antagonist) as first-line treatment for high-risk IHs at a dosage of 2 to 3 mg/kg/day for at least 6 months.

After the first report describing the successful treatment of IHs with oral propranolol in 2008 [9], a large, prospective, multicenter randomized controlled trial including 456 young infants with IHs was conducted in Europe and the Unites States [10]. After that, oral propranolol hydrochloride solution (Hemangeol, Pierre Fabre Pharmaceuticals Inc., Paris, France) was approved by the United States Food and Drug Administration (FDA), the European Medicines Evaluation Agency (EMA) in 2014, and the Korean FDA in 2016, for use in patients with proliferating IHs requiring systemic therapy. Oral propranolol therapy is currently regarded to be the gold standard and first-line treatment for the proliferation of high-risk IHs. Rare but serious side effects of propranolol include bradycardia (0.1%), hypotension (0.1%), hypoglycemia (0.6%), bronchospasm, and bronchial hyperreactivity (0.9–12.9%). Moreover, cardiac side effects can seriously and negatively impact patients. However, few reports [11,12,13] have provided continuous follow-up data regarding cardiac evaluations after propranolol treatment.

Therefore, we prospectively assessed cardiac toxicity in pediatric patients with hemangiomas treated with propranolol for >6 months. Also, we were able to detect congenital heart disease (CHD) by performing an echocardiogram regularly at 6 to 12 months’ follow-up.

## 2. Methods

### 2.1. Study Population

Sixty-four patients diagnosed with IHs and treated with oral propranolol (Hemangeol or Inderal, ANI Pharmaceuticals, Baudette, MN, USA) before 2 years of age at the Department of Pediatrics, Kangbuk Samsung Hospital (Seoul, Republic of Korea) who underwent regular examinations between 2017 and 2021 were included. Patients with a history of CHD or with conflicting information were excluded. Children with chromosomal abnormalities were also excluded. Patients who did not undergo echocardiography tests before and at follow-up were also excluded.

### 2.2. Treatment Protocol

Because most patients with IHs were infants, the initiation of propranolol therapy was performed in an inpatient setting, where propranolol was administered orally at doses of 1 mg/kg/day on the first day of treatment (initiation), 2 mg/kg/day on the second day, and 2–3 mg/kg/day on the third day, divided into 2 daily doses at 9–12 h intervals, during or immediately after feeding to prevent hypoglycemia. Physical examination (pulmonary auscultation, liver palpation, neurodevelopment), glucose, heart rate (HR), blood pressure (BP), and electrocardiography (ECG) monitoring were performed 2 h after each dose to monitor the onset of side effects. Patients were discharged with the maximum tolerable dose of propranolol, and treatment was continued at the outpatient clinic at intervals of 1–2 months.

### 2.3. Efficacy and Safety Assessments (Figure 1)

To assess efficacy, all patients were examined for gross size of hemangiomas using photography every 1–2 months of follow-up during propranolol treatment. Doppler ultrasonography of hemangiomas was performed at diagnosis (i.e., pre-treatment), and at 1 month and 6–12 months of propranolol treatment.

**Figure 1:**
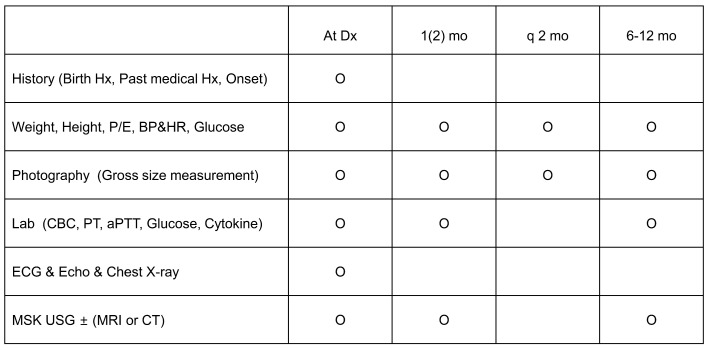
Efficacy and safety assessments before and after oral propranolol treatment in hemangioma. Note: mo, month; Hx, history; BP, blood pressure; HR, heart rate; PE, physical examination; Lab, laboratory; CBC, complete blood count; PT, prothrombin time; aPTT, activated partial thromboplastin time; ECG, electrocardiogram; Echo, echocardiography; MSK, musculoskeletal; USG, ultrasonography; MRI, magnetic resonance imaging; CT, computed tomography.

Safety assessment included the monitoring of common clinical adverse events, physical examinations, growth curves, and development, as well as the monitoring of glucose, BP, and HR, which were performed regularly every 1–2 months during propranolol treatment. Laboratory investigations were performed at initial diagnosis (pre-treatment) and at 1 month and 6–12 months follow-up (post-treatment) during propranolol treatment.

For cardiac evaluations, chest X-ray, ECG, and echocardiography were performed initially (pre-treatment) and at 6–12 months’ follow-up (post-treatment) of propranolol treatment. Some patients with abnormal HR or rhythm underwent Holter monitoring. Cardiac morphology and detailed cardiac function of the left and right ventricles were accurately evaluated using a newly introduced automated three-dimensional (3D) echocardiography system, which has not been commonly used for cardiac evaluation in children. Systolic left ventricular (LV) function was evaluated according to ejection fraction (EF) and fractional shortening (FS) using M-mode echocardiography, mitral annular plane systolic excursion (MAPSE), and global longitudinal strain (GLS). Right ventricle function was evaluated according to peak systolic tissue velocity (S′) and tricuspid annular plane systolic excursion (TAPSE). The parameters of cardiac dysfunction were determined by and taken from the studies [14,15,16].

### 2.4. Statistical Analysis

Statistical analyses were performed using SPSS version 27.0 (IBM Corporation, Armonk, NY, USA). Demographic and clinical data are expressed as percentages or frequencies, median (range [i.e., minimum–maximum]), and mean ± standard deviation (SD), as appropriate. Using a linear mixed model with random effects and a repeated-measures covariance pattern with unstructured covariance within and between patients, significant differences between pre- and post-treatment over time were evaluated. Differences with *p* < 0.05 were considered statistically significant.

## 3. Results

### 3.1. Demographic and Clinical Characteristics

A total of 64 patients (21 male, 43 female) with IHs successfully underwent continuous follow-up cardiac evaluations. The median age at diagnosis was 2 weeks (range, 1 day to 34.3 weeks); the median (minimum–maximum) weight at initial treatment was 7.0 ± 1.8 kg (range, 3.2–11.6 kg). Twelve patients were born preterm (i.e., <37 weeks), and 10 had low birth weight (<2500 g birth weight). The median age at treatment started was 13.6 weeks (range, 2.4–87.9 weeks), the mean longitudinal diameter of hemangioma was 2.8 ± 2.1 cm (range, 0.3–12.0 cm) at diagnosis, and 49 patients had a single lesion, while 15 had multiple lesions. The median duration of treatment was 8 months (range, 6–13 months). After 1 month of oral propranolol treatment, the mean longitudinal diameter of hemangioma was 2.1 ± 2.0 cm (range, 0.0–11.0 cm) and the mean percentage of size decrease was 8.0%. After 1 year of oral propranolol treatment, the average longitudinal diameter of hemangioma was 2.0 ± 2.0 cm (0.0–7.6 cm) and the average percentage of size decrease was 71.8%, which was better when compared with the result (40.5%) of the authors’ previous study [17]. Compliance was good in all 64 patients, and none experienced severe adverse side effects during the treatment, except for bradycardia in 1, hypoglycemia in 2, sleep irritability in 4, and liver enzyme elevation in 3, in whom the propranolol dose was reduced (Table 1).

### 3.2. Echocardiography to Evaluate CHD

Pre-treatment echocardiography revealed CHD in 25 of 64 (39.0%) patients, including three with patent ductus arteriosus (PDA), six with atrial septal defect (ASD), and sixteen with persistent foramen ovale (PFO). On post-treatment echocardiography (6–12 months’ follow-up), three of three PDA, four of six ASD, and 14 of 16 PFOs were closed. All 25 patients with CHD exhibited normal cardiac function and were eligible for oral propranolol therapy (Table 2).

### 3.3. Echocardiography to Assess Cardiac Function

Examination for changes in systolic LV function before and after oral propranolol treatment revealed no statistically significant differences in EF, FS, MAPSE, and 3D EF. EF according to M-mode was in the normal range (>55%) both before (mean, 67.76%) and after (mean, 69.11%) treatment. LVEF according to automated 3D echocardiography, which exhibited closer agreement for LV volume quantification than two-dimensional echocardiography, also demonstrated no statistically significant differences. EF according to 3D echocardiography was in the normal range before (mean, 64.07%) and after (mean, 62.68%) treatment. However, representative LV diastolic function according to tissue Doppler imaging, early (E) and late (A) diastolic mitral inflow velocities, and E/A ratio, together with early diastolic mitral annular velocity e′ (E/e′ ratio) measurements, demonstrated a significant increase after treatment.

For right ventricular (RV) function measurements before and after oral propranolol treatment, peak systolic tissue velocity (S′) and TAPSE were significantly improved after treatment (*p* < 0.05) (Table 2).

### 3.4. ECG and Holter Monitoring to Assess Arrhythmia(s)

None of the 64 patients experienced adverse events, such as arrhythmia, and exhibited no significant changes in HR, PR interval, and QTc, except for QRS duration on ECG during propranolol treatment. Even if QRS durations were statistically different, there were no problems because all indicators were within the normal range (Table 3). Only 2 of 64 patients (3.1%) underwent Holter monitoring for evaluation of abnormal heart rate or rhythm. One patient exhibited sinus arrhythmias on both ECG and Holter monitoring, and the other who exhibited sinus bradycardia and paroxysmal atrial contraction (PAC) on ECG did not exhibit PAC or atrioventricular block on Holter monitoring. No life-threatening arrhythmias were observed.

## 4. Discussion

Results of our study revealed that propranolol administered at 2–3 mg/kg per day (maximum 3 mg/kg/day) was effective and safe without significant cardiac toxicity among IH patients during ≥ 6 months of treatment. Among LV function and RV function measurements, there were no functionally deteriorated indicators; however, diastolic function indicators and GLS of the left ventricle exhibited improvement, and RV function indicators also demonstrated improvement after propranolol treatment. There was a significant increase in post-treatment LV end-diastolic diameter (LVEDD) and LV end-systolic diameter (LVESD) values, reflecting an increase in the volume status of the LV (LVEDD, *p* < 0.001; LVESD, *p* < 0.001) after treatment. After 6–12 months of treatment, there will be a physiological increase in heart size through growth with age. However, there may be an increased preload after treatment, and all LVEDD and LVESD values were in the normal range according to age and were not pathological. Although the right ventricle has a complex geometric shape, in contrast to the ellipsoid shape of the left ventricle, it is difficult to easily assess function using traditional echocardiography. Therefore, the numerical value representing RV function remains unclear. Despite these difficulties, guidelines for the functional assessment of the right ventricle by echocardiography in children, TAPSE, and RV systolic excursion velocity (S′), which are guidelines for functional assessment in children, have improved.

Propranolol is a non-selective antagonist of both β-1 and β-2 adrenergic receptors [18]. Although its exact mechanism of action on hemangiomas is unclear, it has been hypothesized to involve vasoconstriction, the inhibition of angiogenesis, the induction of apoptosis, the inhibition of nitric oxide production, and the regulation of the renin–angiotensin system [19]. Several serious complications have been reported in infants receiving propranolol for His [20]: bradycardia [21], hypotension, prolonged atrioventricular (AV) conduction intensification of AV block [22], bronchospasm, and hypoglycemia or related seizures [23]. However, none of our 64 IH patients exhibited severe toxicities on cardiac function and arrhythmias. Cardiac contraindications for propranolol include sinus bradycardia, sinoatrial block, atrioventricular block, and cardiogenic shock [18,24]. We demonstrated the stability of propranolol in the heart using objective echocardiography and ECG follow-up data. However, there is no definitive protocol for whether cardiac screening should be performed before and/or after oral propranolol treatment except for that of one recent study [13]. Additionally, the need for in-hospital observation during propranolol treatment remains controversial [11]. In some studies [12,25,26], despite no serious contraindications or complications related to propranolol use, cardiac examination and hospitalization of infants and young children should be initiated before treatment. In the study [12], 26% of all patients had CHD and rates that were slightly higher than those reported by Blei et al. [25] but which were significantly higher than other reported results (4–50/1000) [27]. These differences may be related to patient age. A high rate of CHD (39.0%) was observed in the present case. Another study proposed a single ECG screening because CHD may not be identified by murmurs. Therefore, echocardiography is recommended for patients with other cardiac diseases, cyanosis, and HR differences [28]. Some studies have reported that cardiac screening before propranolol treatment should be performed only in patients with indications for CHD [23,29,30,31]. Furthermore, the treatment of hemangioma with propranolol has been well studied; however, there are questions about whether cardiac screening should be performed before the initiation of propranolol treatment [23,25]. Optimal pre-treatment cardiac screening guidelines have not been definitively determined. Therefore, in this study, cardiac examinations before and after propranolol treatment in patients with IHs were evaluated. We prospectively analyzed the incidence of serious short-term and long-term complications at a follow-up of > 6 months after the initial administration of propranolol.

### Strengths and Limitations

The present study was limited by its retrospective design and potential biases, such as undetected confounding factors. Because we studied a limited study population that underwent treatment at a tertiary pediatric center, referral or selection bias cannot be ruled out. The limited number of patients may have restricted the number of complications. Therefore, the impact of pre-treatment diagnosis may not have been fully validated. Additional studies with larger sample sizes may be needed to determine which patients require further analysis, which screenings should be included, and when it is safe and feasible to initiate outpatient propranolol treatment. In addition, because echocardiography is not routinely performed in normal children, the incidence of CHD appears to be high in children with IHs who undergo thorough examination before treatment. However, in reality, all other factors have not been eliminated for the relationship between IHs and CHD. Therefore, it is insufficient to state that there is a relationship between IHs and the incidence of CHD.

## 5. Conclusions

Propranolol was effective and safe, without significant cardiac toxicity, for the treatment of IHs. No severe cardiac toxicity or cardiac contraindications were found for propranolol treatment during routine cardiac screening. However, murmur, cyanosis, or HR differences cannot differentiate all forms of CHD. We demonstrated the stability of the heart during propranolol treatment using objective echocardiography and ECG follow-up data. Initial echocardiography is necessary to diagnose CHD in patients with hemangiomas. Accurate cardiovascular medical history and family history, physical examination, HR, blood pressure, and ECG should be checked routinely before and after treatment to prevent severe cardiac toxicity.

We suggest cardiac toxicity monitoring guidelines. We hope that the results of our study increase the use—or raise awareness—of propranolol treatment for patients with indications for IHs so that attending physicians can treat IHs following appropriate evaluation guidelines with more confidence.

## Figures and Tables

**Table 1:** Demographic and clinical data of infantile hemangioma patients.

Characteristics	Value
Male/Female (n)	21:43
Median gestational age (weeks) ^b^	38.6 (32–41)
Preterm (n) (<37 w)	12
Birth weight (gram) ^a^	3085.4 ± 567.1 (1220–4380)
Low birth weight (n) (below 2500 g)	10
Hemangioma character	
Location (single/mutiple) (n)	49:15
Type (superficial/mixed/deep-seated) (n)	38:11:17
Hemangioma number (n)	1.7 ± 1.0 (1–7)
Hemangioma size	
Longitudinal diameter (cm) ^a^	
Initial	2.8 ± 2.1 (0.3–12.0)
After 1 month	2.1 ± 2.0 (0.0–11.0)
After 1 year	2.0 ± 2.0 (0.0–7.6)
Average percentage of size decrease (%)	
After 1 month	8.0 ± 114.2
After 1 year	71.8 ± 45.7
Treatment	
Median age of diagnosis (weeks) ^b^	2.0 (0.10–34.3)
Median age at initial treatment (weeks) ^b^	13.6 (2.4–87.9)
Median duration of treatment (months) ^b^	8 (6–13)
Median weight at initial treatment (kg)	7.0 ± 1.8 (3.2–11.6)
Cause of treatment (n)	
Risk of functional impairment	10
Local complication (ulceration, bleeding)	3
Risk of aesthetic impairment	51
Side effect patient (n)	11
Side effect character (n)	
Hypotension	0
Bradycardia	1
Hypoglycemia	2
Insomnia	4
Elevated liver enzyme	3

^a^ Categorical variables were expressed as frequency distributions and continuous variables as mean values ± standard error of the mean. ^b^ All variables described statistics median values (minimum–maximum). A *p*-value < 0.05 was considered significant.

**Table 2:** Echocardiography pre- and post-treatment in infantile hemangioma patients.

Echocardiography	Pre	Post	Coefficient (95% Confidence Interval)	*p* Value
Functional study				
Functional study				
LV function				
2D EF (%)	67.76 ± 4.03	69.11 ± 5.17	−1.342 (−3.23 to 0.54)	0.158
2D FS (%)	35.76 ± 3.21	37.37 ± 4.03	−1.61 (−3.11 to −0.11)	0.036
LV mass index (g/m^2^)	46.21 ± 8.54	46.19 ± 9.25	0.022 (−3.13 to 3.17)	0.989
IVS thickness (mm)	4.18 ± 0.75	4.14 ± 0.74	0.048 (−0.27 to 0.37)	0.765
LVPW thickness (mm)	3.79 ± 0.5	3.81 ± 0.71	−0.022 (−0.26 to 0.22)	0.853
LVEDD (mm)	23.96 ± 4.01	28.23 ± 3.7	−4.266 (−5.13 to −3.4)	<0.001
LVESD (mm)	15.31 ± 2.46	17.67 ± 2.91	−2.363 (−3.07 to −1.65)	<0.001
E/A ratio	4.18 ± 0.75	4.14 ± 0.74	−0.209 (−0.33 to −0.09)	0.002
E/e′	3.79 ± 0.5	3.81 ± 0.71	1.167 (0.37 to 1.97)	0.005
MAPSE (mm)	23.96 ± 4.01	28.23 ± 3.7	−0.059 (−0.73 to 0.61)	0.859
GLS	15.31 ± 2.46	17.67 ± 2.91	1.162 (0.05 to 2.27)	0.040
3D EF (%)	64.07 ± 4.69	62.68 ± 4.05	−1.393 (−3.421 to 0.635)	0.170
RV function				
TV e′ (m/s)	9.92 ± 2.87	9.98 ± 2	−0.003 (-0.02 to 0.01)	0.662
TV a′ (m/s)	−19.19 ± 2.2	−20.35 ± 2.38	0.017 (0.001 to 0.03)	0.035
TV s′ (m/s)	10.56 ± 2.3	9.39 ± 1.2	−0.012 (−0.02 to −0.005)	0.002
TAPSE (mm)	14.08 ± 2.41	15.36 ± 2.31	1.283 (0.407–2.159)	0.006
Morphologic study—congenital heart disease (n)				
Atrial septal defect	6	2		
Persistent foramen ovale	16	2		
Patent ductus arteriosus	3	0		

a′, peak late diastolic velocity; e′, peak early diastolic velocity; 3D, three dimensional; EF, ejection fraction; E/A, early/late diastolic inflow velocity; E/e′, ratio of the E wave/e′; FS, fractional shortening; GLS, global longitudinal strain; IVS, interventricular septum; LV, left ventricle; LVEDD, left ventricular end-diastolic diameter; LVESD, left ventricular end-systolic diameter; LVPW, left ventricular posterior wall; MAPSE, mitral annular plane systolic excursion; RV, right ventricle; s′, peak systolic tissue velocity; TAPSE, tricuspid annular plane systolic excursion. All variables were expressed as frequency distributions and continuous variables as mean values ± standard error of the mean. A *p* value < 0.05 was considered significant.

**Table 3:** Electrocardiogram pre- and post-treatment in patients with infantile hemangiomas.

	Pre	Post	Coefficient (95% CI)	*p* Value
Heart rate	120.15 ± 23.73	111.56 ± 22.47	8.58 (−0.55 to 17.73)	0.065
PR interval	0.12 ± 0.02	0.12 ± 0.02	−0.01 (−0.01 to 0.004)	0.263
QRS duration	0.07 ± 0.01	0.07 ± 0.02	−0.01 (−0.01 to −0.001)	0.018
QTc	427.44 ± 20.31	425.74 ± 17.75	1.70 (−6.93 to 10.34)	0.690

All variables were expressed as frequency distributions and continuous variables as mean values ± standard error of the mean. A *p* value < 0.05 was considered significant.

## Data Availability

The datasets used and analyzed during the current study are available from the corresponding author upon reasonable request.
